# Hekun decoction versus Femoston for women with amnestic mild cognitive impairment in early menopause: a randomized, three-arm, double-blind clinical trial

**DOI:** 10.3389/fneur.2025.1610562

**Published:** 2025-09-12

**Authors:** Ziqi Zhai, Guangyao Lin, Siru Chen, Huicong Liu, Rui Yuan, Xianwei Gao, Xiaorong Ni, Mingjie Shen, Haiying Fan, Wenjun Wang, Yan Zhao, Yan Wu, Chao Gu, Chao Cong, Shan Xiao, Yue Zhang, Jingchang Zhao, Jiayao Xu, Ye Wang, Jie Chen, Yishuang Chen, Yan Zong, Qianjue Tang, Shengnan Li, Zhijie Zhang, Lianwei Xu

**Affiliations:** ^1^Department of Gynecology, Longhua Hospital, Shanghai University of Traditional Chinese Medicine, Shanghai, China; ^2^School of Public Health, Fudan University, Shanghai, China; ^3^Shanghai Municipal Hospital of Traditional Chinese Medicine, Shanghai University of Traditional Chinese Medicine, Shanghai, China; ^4^Shuguang Hospital Affiliated to Shanghai University of Traditional Chinese Medicine, Shanghai, China; ^5^Fengxian Hospital Affiliated to Shanghai University of Traditional Chinese Medicine, Shanghai, China; ^6^Obstetrics and Gynecology Hospital of Fudan University, Shanghai, China; ^7^Shanghai Baoshan District Hospital of Integrated Traditional Chinese and Western Medicine, Shanghai, China

**Keywords:** amnestic mild cognitive impairment, Hekun decoction, early menopausal women, Alzheimer’s disease, clinical trial

## Abstract

**Background:**

Amnestic mild cognitive impairment (aMCI), a prodromal stage of Alzheimer’s disease (AD), carries a high risk of progression to dementia. However, few clinical trials have focused on interventions to delay this progression. Hekun Decoction, an herbal-based oral medicine, has shown potential in improving memory loss during early menopause. Here we performed a randomized controlled trial to evaluate the efficacy and safety of Hekun Decoction in women with aMCI.

**Methods:**

This prospective, randomized, three-arm, double-blind clinical trial enrolled women aged 40–60 years with aMCI during early menopause. Participants were randomized to Hekun Decoction, Femoston, or placebo for 24 weeks. The primary outcome was the change in the Montreal Cognitive Assessment (MoCA) score at 0 and 24 weeks. The secondary outcomes included the Menopause Rating Scale (MRS), Modified Kupperman Index (KI), and Insomnia Severity Index (ISI), along with adverse events.

**Results:**

Between October 2021 and February 2024, a total of 292 patients were randomized to Hekun Decoction (*n* = 98), Femoston (*n* = 98), and placebo (*n* = 96). After 24 weeks, both Hekun Decoction (MD = 3.18, 95% CI [2.44–3.92]) and Femoston (MD = 3.67, 95% CI [2.93–4.42]) were more effective than placebo in improving MoCA scores. Meanwhile, better outcomes were observed in the Hekun Decoction group and Femoston group compared with the placebo group for MRS (MD = −5.87, 95% CI [−7.02, −4.71], and MD = −6.01, 95% CI [−7.02, −5.01]), KI (MD = −6.74, 95% CI [−8.16, −5.33], and MD = −6.93, 95% CI [−8.27, −5.59]), and ISI (MD = −6.53, 95% CI [−7.66, −5.39], and MD = −6.51, 95% CI [−7.58, −5.43]). As for the adverse events, no cases of abdominal distension, pain, breast pain, or abnormal uterine bleeding were observed in the Hekun Decoction group.

**Conclusion:**

In women with aMCI during early menopause, Hekun Decoction demonstrated non-inferior efficacy to Femoston in improving cognitive function over 24 weeks, with a favorable safety profile. Notably, women in the Hekun Decoction group showed fewer adverse events compared with Femoston. However, further trials with longer follow-up periods are needed to confirm the efficacy of Hekun Decoction in women with aMCI.

**Clinical trial registration:**

http://chictr.org.cn, ChiCTR2000036772.

## Introduction

Mild cognitive impairment (MCI) is an intermediate state between normal aging and Alzheimer’s disease (AD), manifested as a progressive decline in memory or other cognitive functions that does not affect the ability to perform daily living activities and does not meet the diagnostic criteria for dementia (1). Amnestic mild cognitive impairment (aMCI), one of the subtypes of MCI, is characterized by memory impairment, accounting for 66.5% of all MCI cases, and is a prodromal stage of AD (2, 3). Annual progression rates from aMCI to AD range from 10 to 15% (4–6), exceeding rates observed in cognitively normal populations (7). However, Among patients with aMCI, women tend to show more severe cognitive decline and faster progression to AD (8). Approximately 40.6–56% of pre- and post-menopausal women have aMCI manifestations such as memory loss, which may be related to the precipitous decline in estrogen levels during menopause (9–11). Notably, a recent statistic revealed that menopausal hormone therapy (MHT) initiated within 5 years postmenopause was associated with a 30% reduction in AD risk; however, the risk of AD will also be increased in women who underwent MHT more than 5 years after menopause (12, 13). In other words, a ‘window of opportunity’ exists for MHT of aMCI to prevent AD.

Unfortunately, several adverse and unpredictable events like venous thromboembolism, breast cancer, and stroke caused by hormonal therapy have restricted its extensive use in clinical (14–16). Meanwhile, current treatment approaches have shown limited efficacy in slowing down the disease progression (13). Recently, a considerable number of clinicians are striving to explore novel therapy to inform clinical practice in the treatment of AD (17). Traditional Chinese medicine (TCM) has been widely practiced in neurodegenerative disorders for thousands of years with emerging scientific evidence (18, 19). Therefore, TCM has been considered as a beneficial intervention for improving cognitive functions and life quality in people with neurodegenerative disorders, which has been proven to involve diverse therapeutic mechanisms (20–22). However, as few large trials have compared TCM with placebo treatment to supporting these promising TCM as effective candidate drugs, there was a clear need for a large rigorous trial investigating the effectiveness of TCM relative to placebo treatment.

Hekun Decoction has demonstrated potential for clinical application in the management of aMCI, with accumulating experience supporting its use in practice. Our previous clinical observation has shown that Hekun Decoction improved several clinical symptoms of perimenopausal women, including memory loss, insomnia, dreaminess, and inattention. Although these findings led to the hypothesis that Hekun Decoction might be an effective intervention for women with aMCI, clinical evidence supporting the use of Hekun Decoction to ameliorate cognitive function for women with aMCI during early menopause remains unclear. Therefore, we undertook a randomized controlled, three-arm, double-blind clinical trial to address the following questions: (1) Is Hekun Decoction an effective intervention for cognitive improvement in women with aMCI? (2) Is Hekun Decoction safe and well tolerated for clinical use?

## Methods

### Study design

This was a randomized controlled, three-arm, double-blind trial conducted in six hospitals between October 2021 and December 2023, including I. Longhua Hospital, Shanghai University of Traditional Chinese Medicine; II. Shuguang Hospital affiliated to Shanghai University of Traditional Chinese Medicine; III. Fengxian Hospital affiliated to Shanghai University of Traditional Chinese Medicine; IV. Obstetrics and Gynecology Hospital of Fudan University; V. Shanghai Baoshan District Hospital of Integrated Traditional Chinese and Western Medicine, and VI. Shanghai Municipal Hospital of Traditional Chinese Medicine, Shanghai University of Traditional Chinese Medicine. The trial protocol is also available at chictr.org.cn (ChiCTR2000036772). The trial was approved by Ethics Committee of Longhua Hospital, Shanghai University of Traditional Chinese Medicine (2021LCSY046), and conducted in accordance with the Declaration of Helsinki, the International Conference on Harmonization Good Clinical Practice, along with Chinese regulations. Written informed consent was obtained from all patients or legally authorized representatives.

### Participants

Eligible women were aged 40–60 years with early menopause, aMCI according to internationally recognized diagnostic criteria (23) (I, complaints of memory impairment from patient or family members; II, complaints of impairment in one or more cognitive domains, including memory, executive function, attention, language, and visuospatial skills; III, normal activities of daily living; IV, the cognitive impairment in memory did not meet diagnostic criteria for dementia), and diagnosed with kidney deficiency syndrome according to TCM criteria, along with participants were able to undergo neuropsychological tests.

Participants were excluded if they were diagnosed with dementia according to the diagnosis of Mini-Mental State Examination (MMSE) (>24 scores for subjects with secondary education or above, >20 scores for elementary school or less, and >17 scores for those with no formal education) (20, 24), had a history of alcohol or drug abuse or addiction, had severe depression or severe anxiety or a history of schizophrenia or other psychiatric disorders, had a known or suspected history of allergy to the test drug and its excipients, had contraindications to hormone therapy (such as breast cancer, endometrial cancer, deep vein thrombosis, liver abnormalities, and so forth). Complete inclusion and exclusion criteria are provided in Supplementary material S1.

### Randomization and masking

After participants completed the baseline assessment, the randomization was performed by an independent, unmasked statistician. In this study, we used multicenter randomization for competitive enrollment, and the randomized list method was used to randomly assign eligible participants to the Hekun Decoction group, Femoston group, and the placebo group (1:1:1 ratio of allocation). The drugs in the three groups were identical in taste and appearance. Allocation was concealed, and participants were linked to study groups by identification numbers printed on drug labels. Documentation of the list of randomized subgroups to which the identification numbers were linked was visible only to the producers, and these supplements were maintained by two individuals who otherwise did not participate in the study.

### Sample size calculation

The study was designed as a non-inferiority trial focusing on the primary efficacy endpoint: the change in MoCA from baseline to 24 weeks. We set the total type I error to 0.05 and the test power to 0.9. Based on the results of our previous clinical observation, the smallest difference in MoCA scores between any two groups was set to 2, the standard deviation was 2.5, and the assumed dropout rate was 15%. Considering multiple comparisons, the required sample size calculated by the PASS 2020 software was 96 cases per group, and 288 cases were needed.

### Intervention

In the Hekun Decoction group, the treatment comprised Hekun Decoction, which consists of 13 traditional Chinese medicines (Manufactured by the Neo-Green Pharmaceutical Co., Ltd. Sichuan, China) and Femoston simulant (containing estradiol and dydrogesterone) (Nanjing Haina Pharmaceutical Co., production lot number: 210630111). In the Femoston group, the treatment comprised Hekun Decoction simulant which contains 10% dose of Hekun Decoction (Manufactured by the Neo-Green Pharmaceutical Co., Ltd. Sichuan, China) and Femoston (Abbott, production lot number: 210630111). In the placebo group, the treatment comprised Hekun Decoction simulant plus Femoston simulant.

Pharmacists selected appropriately sized drugs, where possible, matched color, taste, and appearance of drugs to the Hekun Decoction or Femoston. The intervention duration was 24 weeks. All participants were required to continue taking medication after enrollment. Oral Hekun Decoction or Hekun Decoction simulant was administered once in the morning and once in the afternoon, respectively. The Femoston is taken once a day continuously. The composition of Hekun Decoction is provided in Supplementary material S2.

### Study outcomes

The primary efficacy endpoint was global cognition assessed by the Montreal Cognitive Assessment (MoCA) between baseline (0 week) and 24 weeks. The MoCA scores range from 0 to 30, with higher scores indicating better cognitive function (25). Secondary endpoints included evaluation of menopausal symptoms, sleep quality assessment tests between baseline (0 week) and 24 weeks. Evaluation of menopausal symptoms included the Menopause Rating Scale (MRS) and the Modified Kupperman Index Scale (KI). The sleep quality was evaluated by Insomnia Severity Index (ISI).

### Statistical analysis

Two investigators recorded all data in an Excel database, and SPSS software (version 27.0) was used for statistical analysis of the research data. The measurement data were expressed using mean±standard deviation (X ± s), and the counting data were described using frequency and component ratios. Univariate analysis of variance was used to compare the age, height, weight, and body mass index (BMI) of all enrolled patients. The Fisher–Freeman–Halton test was used to analyze cognition-related risk factors, such as education level, occupational type differences, economic status, and living habits of all enrolled patients. Using generalized estimation equations with corresponding baseline scores as covariates, MRS, and KI scores from baseline to 24 weeks after treatment. Univariate ANOVA and Dunnett’s test were used to perform multiple post-treatment comparisons among the three groups if the variances were homogeneous. If the variance was not uniform, Tambane’s T2 test was used. After that, the corresponding baseline scores were analyzed again using generalized estimation equations as covariates. *p* < 0.05 was the standard for statistical differences.

## Results

### Participant characteristics

**Figure 1:**
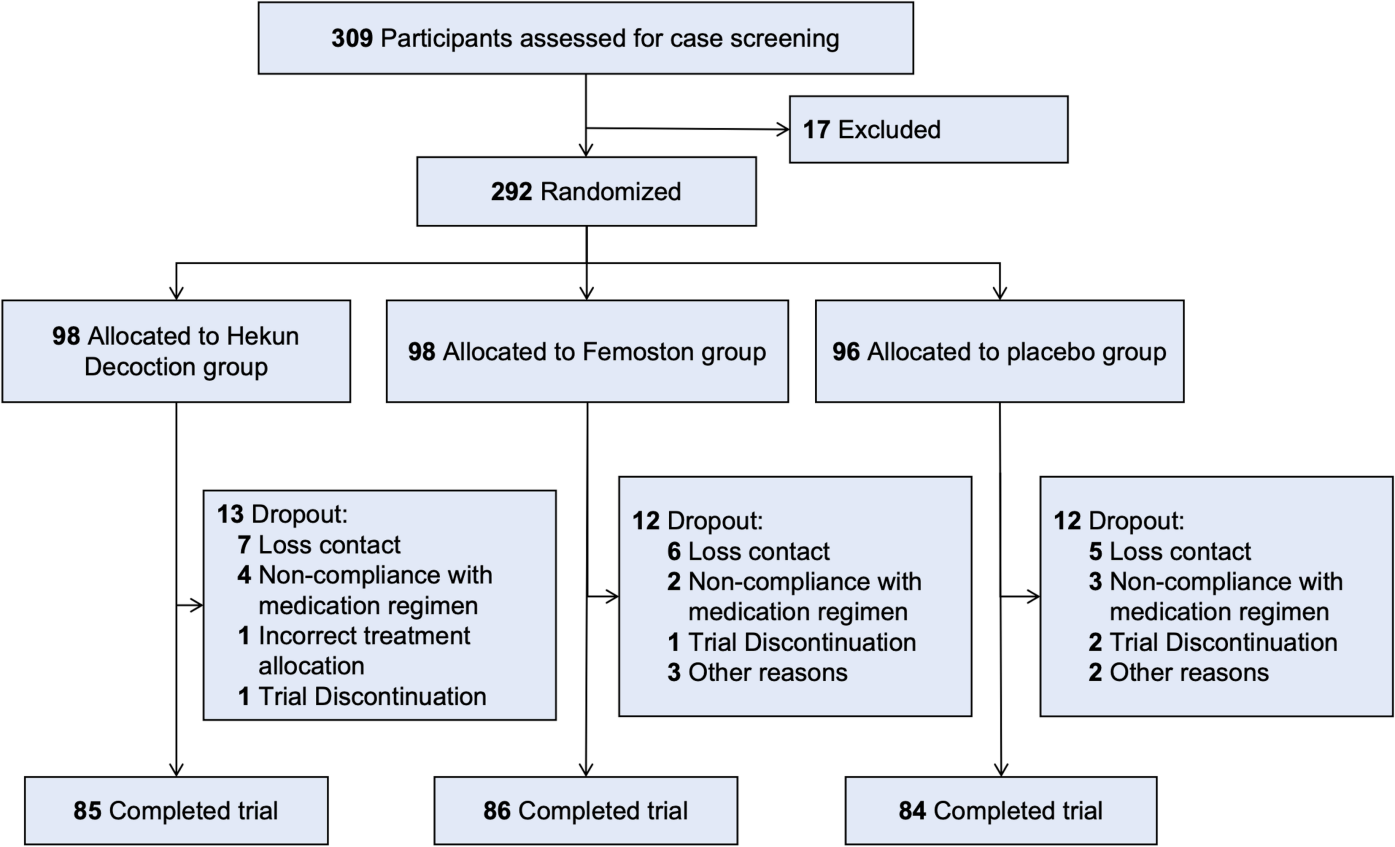
Flow of participants in the trial.

Three hundred and nine participants were enrolled in the trial from 6 centers in Shanghai, China to be screened for eligibility, and 292 participants were randomly assigned to treatment (98 women in Hekun Decoction group, 98 women also in Femoston group, and 96 women in placebo group). Complete information on the primary efficacy endpoint was obtained for 255 (91.44%) of the randomized population (Figure 1). The detailed reasons for withdrawal and exclusion of participants in this study were shown in Supplementary material S3.

Overall, the age of study participants was 40–60 years. 40.07% of the eligible participants received middle school education (117/292), and 68.49% are predominantly mental labor (200/292), but 67.47% of the participants did not have a reading habit (197/292). Besides, MMSE among Hekun Decoction group (24.49 ± 2.36), Femoston group (24.69 ± 2.34), and placebo group (24.80 ± 2.81) showed no significant difference. The participants’ baseline demographic and clinical characteristics are summarized in Table 1.

**Table 1:** Participant characteristics.

Variables	Hekun decoction (*n* = 98)	Femoston group (*n* = 98)	Placebo group (*n* = 96)	All (*n* = 292)
Age (year)	51.64 ± 4.04	52.20 ± 4.09	52.47 ± 3.90	52.10 ± 4.01
Height (cm)	158.87 ± 4.33	159.74 ± 4.24	159.58 ± 4.68	159.40 ± 4.42
Weight (kg)	59.63 ± 7.26	60.58 ± 7.34	60.48 ± 7.69	60.23 ± 7.42
BMI (kg/m^2^)	23.63 ± 2.77	23.74 ± 2.78	23.74 ± 2.72	23.70 ± 2.75
Education level—No. (%)				
Elementary school and below	28 (28.6)	14 (14.3)	27 (28.1)	69 (23.6)
Middle school	37 (37.8)	45 (45.9)	35 (36.5)	117 (40.1)
High school	11 (11.2)	21 (21.4)	15 (15.6)	47 (16.1)
Undergraduate and above	22 (22.4)	18 (18.4)	19 (19.8)	59 (20.2)
Type of occupation—No. (%)				
Physical labor	34 (34.7)	35 (35.7)	23 (24)	92 (31.5)
Mental labor	64 (65.3)	63 (64.3)	73 (76.0)	200 (68.5)
Reading habits—No. (%)				
None	64 (65.3)	68 (69.4)	65 (67.7)	197 (67.5)
Less than 30 min per day	11 (11.2)	10 (10.2)	11 (11.5)	32 (11.0)
30 min to 1 h per day	14 (14.3)	15 (15.3)	13 (13.5)	42 (14.4)
1–3 h per day	7 (7.1)	4 (4.1)	6 (6.3)	17 (5.8)
More than 3 h per day	2 (2.0)	1 (1.0)	1 (1.0)	4 (1.4)
Exercise—No. (%)				
No	54 (55.1)	51 (52.0)	61 (63.5)	166 (56.8)
Yes	44 (44.9)	47 (48.0)	35 (36.5)	126 (43.2)
Dietary preferences—No. (%)				
Animal-based diet	6 (6.1)	5 (5.1)	10 (10.4)	21 (7.2)
Plant-based diet	15 (15.3)	11 (11.2)	21 (21.9)	47 (16.1)
Dietary structure with a balance of animal and plant foods	77 (78.6)	82 (83.7)	65 (67.7)	224 (76.7)
Monthly income—No. (%)				
Below 2,000 Yuan	3 (3.1)	3 (3.1)	1 (1.0)	7 (2.4)
2,001 to 5,000 Yuan	61 (62.1)	62 (63.3)	65 (67.7)	188 (64.4)
5,001 to 10,000 Yuan	22 (22.4)	26 (26.5)	21 (21.9)	69 (23.6)
10,001 yuan and above	12 (12.2)	7 (7.1)	9 (9.4)	28 (9.6)

### Primary efficacy outcome

#### MoCA

For the cognitive function, the mean MoCA value at baseline was 22.89 ± 2.58 in the Hekun Decoction group and 22.98 ± 2.42 in the Femoston group. After treatment for 24 weeks, the MoCA scores increased to 25.41 ± 2.84 in the Hekun Decoction group and 26.03 ± 2.44 in the Femoston group. Whereas, it is not observed that participants received placebo treatment (22.67 ± 2.86) have increase in MoCA compared with baseline (22.07 ± 2.31).

Meanwhile, the cognitive function on women with aMCI was also enhanced after intervention with Hekun Decoction (MD = 3.18, 95% CI [2.44–3.92], *p* < 0.01) as well as Femoston (MD = 3.67, 95% CI [2.93, 4.42], *p* < 0.01) compared to placebo group at 24 weeks (Table 2).

**Table 2:** Clinical outcomes in the three groups.

Variables	Hekun decoction	Femoston	Placebo	Hekun decoction *vs*. femoston		Hekun decoction *vs.* placebo		Femoston *vs.* placebo	
	(*n* = 85)	(*n* = 86)	(*n* = 84)	MD (95% CI)^a^	*p* ^b^	MD (95% CI)^a^	*p* ^b^	MD (95% CI)^a^	*p* ^b^
MoCA									
Baseline	22.85 (2.59)	22.97 (2.43)	22.67 (2.86)	−0.50 (−1.12, 0.13)	0.16	3.18 (2.44, 3.92)	<0.01	3.67 (2.93, 4.42)	<0.01
24 weeks	25.41 (2.84)	26.03 (2.44)	22.07 (2.31)						
MRS									
Baseline	11.46 (3.91)	11.17 (3.37)	11.00 (4.00)	0.15 (−0.86, 1.16)	0.76	−5.87 (−7.02, −4.71)	<0.01	−6.02 (−7.02, −5.02)	<0.01
24 weeks	6.94 (2.99)	6.51 (3.04)	12.30 (4.86)						
KI									
Baseline	16.18 (4.14)	16.03 (3.47)	16.01 (5.72)	0.18 (−1.10, 1.47)	0.56	−6.74 (−8.16, −5.33)	<0.01	−6.93 (−8.27, −5.59)	<0.01
24 weeks	10.22 (3.99)	9.85 (4.18)	16.74 (5.12)						
ISI									
Baseline	13.39 (4.39)	13.19 (4.19)	12.67 (4.83)	−0.02 (−1.04, 0.99)	≈1.00	−6.53 (−7.66, −5.39)	<0.01	−6.51 (−7.58, −5.43)	<0.01
24 weeks	7.76 (3.89)	7.66 (3.67)	13.58 (6.09)						

a Mean difference (95% CI) of the difference between two groups from baseline to 24 weeks after treatment (after–before).

b Pairwise comparisons of mean differences using Wilcoxon rank sum test with continuity correction (Bonferroni method). Data are mean (SD). Higher scores represent worse outcomes for all measures. MoCA, Montreal Cognitive Assessment; MRS, Menopause Rating Scale; KI, Modified Kupperman Index Scale; ISI, Insomnia Severity Index. Montreal Cognitive Assessment (MoCA) scores range from 0 to 30, with lower scores indicating greater cognitive impairment. MRS scores range from 0 to 44, with higher scores indicating more severe menopausal symptoms. KI scores range from 0 to 63, with higher scores indicating more severe menopausal symptoms. ISI scores range from 0 to 28; higher scores indicate more severe symptoms.

### Secondary outcomes

Table 2 summarizes the results of analyses for secondary endpoints. First, the change from baseline to 24 weeks in menopausal symptoms, determined using the MRS was statistically different between baseline (11.46 ± 3.91) and 24 weeks (6.94 ± 2.99) in Hekun Decoction group. A remarkably statistical amelioration was also observed regarding the outcomes of MRS before (11.17 ± 3.37) and after (6.51 ± 3.04) intervention with Femoston, but there was no improvement after treatment with placebo (12.30 ± 4.86) compared with baseline (11.00 ± 4.00). Simultaneously, our results also showed sufficient evidence of Hekun Decoction (MD = −5.87, [−7.02, −4.71], *p* < 0.01) and Femoston (MD = −6.02, [−7.02, −5.02], *p* < 0.01) intervention had similar efficacy between groups in improving MRS compared with those administered placebo. Still, there was no difference on MRS between Hekun Decoction and Femoston treatment.

Second, concerning the KI, evidence from our studies suggested that Hekun Decoction had a notable decrease on KI (10.22 ± 3.99) compared with baseline (16.18 ± 4.14). There was evidence of a substantial improvement in KI with Femoston treatment (9.85 ± 4.18) compared to baseline (16.03 ± 3.47). However, it was noteworthy that there was no striking amelioration after treatment with placebo (16.74 ± 5.12) compared to baseline (16.01 ± 5.72). What’s more, this trial also suggested that Hekun Decoction (MD = −6.74, [−8.16, −5.33], *p* < 0.01) and Femoston (MD = −6.93, [−8.27, −5.59], *p* < 0.01) showed comparable efficacy compared to placebo group.

Third, ISI was recorded in three groups. The pooled results showed significant decreasein ISI after Hekun Decoction treatment for 24 weeks (7.76 ± 3.89) compared to baseline (13.39 ± 4.39). It was noteworthy that ISI was also significantly diminished from 13.19 ± 4.19 before treatment to 7.66 ± 3.67 after treatment with Femoston. However, no statistically significant difference before (12.67 ± 4.83) and after (13.58 ± 6.09) treatment with placebo. Additionally, pooling of these data indicated a striking improvement between Hekun Decoction (MD = −6.53, [−7.66, −5.39], *p* < 0.01) and Femoston (MD = −6.51, [−7.58, −5.43], *p* < 0.01) treatment in ISI compared to placebo group.

### Adverse events

Overall, 32 participants (10.96%) experienced at least 1 adverse events. Among them, 4 participants (4.08%) were in the Hekun Decoction group, 19 participants (19.39%) were in the Femoston group, and 9 (9.38%) participants were in the placebo group during the 24-week follow-up period. Collectively, Hekun Decoction demonstrated a favorable safety profile, with fewer adverse effects compared to the Femoston group (Supplementary material S4).

## Discussion

Menopausal women tend to experience a range of perimenopausal symptoms such as hot flashes, memory loss, palpitations, insomnia and so forth. Approximately 76.54% of women experience memory decline, and the trend of memory loss occurrence increases from the early stage of the menopausal transition and reaches a peak in the late stage of the menopausal transition (26). In our clinical practice, though a considerable number of menopausal women seek Chinese medicine for the main complaint of poor memory, patients’ memorization ability is rarely assessed by clinicians. The MoCA which was adopted by our study is a cognitive screening tool that aims to differentiate healthy cognitive aging from aMCI (27). Therefore, our results were more accurate to estimate the aMCI situation. In addition, the MMSE is widely used to measure cognitive function in patients with AD. Therefore, we only used the MMSE scale to assess cognitive ability and exclude patients with AD.

This study observed that Hekun Decoction could effectively improve cognitive function in early menopausal women with aMCI. Compared with that of the placebo group, the MoCA score of the Hekun Decoction group was higher, which indicated Hekun Decoction has positive effects on improving cognitive function. Besides, our study adopted Femoston to enhance the cognitive ability of women in early menopause. Femoston, a widely used MHT, exerts neuroprotective effects through facilitating higher cognitive functions by mediating estrogen receptors α (ER α), ER β, and G protein-coupled estrogen receptor 1 (GPER1), along with activating Akt, LIMK, TrkB neurotrophin receptor in the cortex and hippocampus (28). Meanwhile, a meta-analysis confirmed MHT efficacy in reducing cognitive decline among postmenopausal women (29). Therefore, this study selected Femoston as a comparator, and the findings suggested that Femoston could increase the MoCA score, which was also consistent with previous research results using MHT within 5 years of menopause (30). Comparing the MoCA between Hekun Decoction and Femoston groups, no significant difference in total scales scores was observed in the cognitive function after treatment, which throws light on the fact that Hekun Decoction and Femoston have valuable significance in improving aMCI compared to placebo group.

Especially, our study recruited women with early menopausal who are at a high risk “forgetfulness,” one of the main characteristic of aMCI (31). As the TCM theory put, early menopausal women belong to the liver-kidney deficiency syndrome, followed by the corresponding cognitive decline symptoms. Therefore, Hekun Decoction with the treatment of tonifying the kidneys and filling up the vital essence can provide good therapeutic effects. Also, it embodies the concept of “preventive treatment of disease” in Chinese medicine, if women with aMCI at the stage of early menopause received effective intervention. Besides, MHT studies have also shown a role for early menopausal hormone intervention in preventing AD development and progression (30).

In addition to improving cognitive function, compared with the placebo group, Hekun Decoction and Femoston could significantly relieve the clinical symptoms of the menopausal syndrome in women with early menopausal aMCI, such as hot flush sweating, insomnia, fatigue. Interestingly, the symptoms of fatigue were considerably improved with the treatment of Hekun Decoction compared to Femoston. Moreover, we further investigated whether sleep quality was correlated with cognitive ability, and the results indicated that ISI were associated with MoCA. Currently, a great number of articles also demonstrated that for aged women, habitual sleep duration forecasts the future potential risk for cognitive impairments such as dementia; meanwhile, another study implied that stable sleep of at least 7 h/night improves response inhibition and working memory in healthy adults (32, 33), which was consistent with our findings. A recent study reported a correlation between fatigue and sleep disturbances, suggesting that alleviating fatigue may improve sleep quantity. Statistically, we also identified correlations between ISI, MRS, KI, and MoCA according to the Pearson correlation analysis. Therefore, the use of Hekun Decoction to interfere with MCI in early menopause was associated with improvements in sleep quality, relieve menopausal symptoms such as fatigue, improve cognitive functions, and may delay the development of AD. Meanwhile, this trial also demonstrated that 24 weeks use of Hekun Decoction is safe, and the adverse events in the Hekun Decoction were mild, transient, and disappeared independently.

This study had some limitations. First, although cognitive decline typically progresses slowly, and short-term outcomes may not reflect long-term benefits or risks, this study was limited to 24 weeks. Therefore, further long-term observation is needed to determine whether cognitive function fluctuates or worsens in women with early menopausal aMCI following short-term medication use. Second, larger-scale multicenter RCTs are needed to investigate whether more prolonged use of Hekun Decoction treatment will yield results that maintain the good outcomes already achieved and delay or reduce the occurrence of AD. Third, the clinical study was performed exclusively with menopausal women at several hospitals in Shanghai, China, thereby the generalizability of these findings to other hospital settings in other regions is not clear. Fourth, although this study adopted the internationally recognized MoCA to evaluate treatment efficacy, there is a lack of specificity measures to objectively assess aMCI, which might not reflect the actual efficacy after intervention with Hekun Decoction and Femoston. Thereby, a great deal of effort is needed to establish a well-recognized rules to evaluate‌ treatment efficacy for aMCI. Also, future clinical trials with extended follow-up periods, involving diverse regions and ethnic participants, are required to confirm the generalizability of Hekun Decoction.

## Conclusion

In women with aMCI in early menopause, Hekun Decoction significantly improved cognitive function at 24 weeks, demonstrating non-inferiority compared with Femoston. Notably, Hekun Decoction and Femoston had a better impacts on ameliorating menopausal syndromed compared to placebo. However, further trials with longer follow-up periods (at least 12 months) are needed to confirm the efficacy of Hekun Decoction in women with aMCI.

## Data Availability

The original contributions presented in the study are included in the article/Supplementary material, further inquiries can be directed to the corresponding authors.
