# Supplementary material for: Hekun decoction versus Femoston for women with amnestic mild cognitive impairment in early menopause: a randomized, three-arm, double-blind clinical trial
Source: Front Neurol. 2025 Sep 12;16:1610562. doi: 10.3389/fneur.2025.1610562 (PMC12463637; doi:10.3389/fneur.2025.1610562)
Supplement: Supplementary file 1 [file Table_1.DOCX]

# **Supplementary material 1：**

# Inclusion Criteria And Exclusion Criteria

Inclusion Criteria:

- Age 40-60 years old;
- Meet the Western diagnostic criteria for early menopause and aMCI;
- Patients with kidney essence deficiency syndrome who meet the standard of TCM syndrome differentiation;
- The ability to take neuropsychological tests;
- Volunteer to participate in this study and sign the informed consent.

Exclusion Criteria:

- Patients with dementia (Alzheimer's disease, AD);
- Patients with serious diseases such as cardiovascular and cerebrovascular, liver, kidney or hematopoietic system;
- The presence or suspected history of breast cancer, estrogen-dependent malignancies (such as endometrial cancer), progesterone dependent tumors;
- Patients with past idiopathic or existing venous thromboembolism (deep venous embolism, pulmonary embolism);
- Active or recent arterial thromboembolic disease (such as angina pectoris, myocardial infarction);
- Those suffering from porphyria;
- The presence of unexplained reproductive tract bleeding;
- Patients with long endometrial hyperplasia and untreated;
- Suspected or true pregnancy;
- People with a history of alcohol, drug abuse or addiction;
- A history of severe depression or severe anxiety or schizophrenia or other mental illness;
- Patients with Parkinson's disease, multiple sclerosis, epilepsy and other neurological diseases accompanied by persistent neurological deficits or known structural brain abnormalities
- Inability to cooperate with researchers;
- Patients who have used prohibited drugs (cognitive-improving drugs, hormone drugs) for the past 3 months, or participated in other clinical trials;
- Known or suspected allergic history to the experimental drug and its excipients.
