# Supplementary material for: Hekun decoction versus Femoston for women with amnestic mild cognitive impairment in early menopause: a randomized, three-arm, double-blind clinical trial
Source: Front Neurol. 2025 Sep 12;16:1610562. doi: 10.3389/fneur.2025.1610562 (PMC12463637; doi:10.3389/fneur.2025.1610562)
Supplement: Supplementary file 2 [file Table_2.DOCX]

| **Chinese name** | **Latin name** | **Amount (g)** | **Lot number** |
| --- | --- | --- | --- |
| Sheng Di Huang | Radix Rehmanniae Praeparata | 12 | 21020061 |
| Bai Shao | Sautéed Paeonia lactiflora | 15 | 21100295 |
| Ba Ji Tian | Morinda citrifolia | 9 | 21100665 |
| Xian ling Pi | Epimedium | 15 | 21100266 |
| Huang Bai | Cortex Phellodendri Chinensis | 9 | 20120089 |
| Chai Hu | Radix Bupleuri | 9 | 20120142 |
| Long Gu | Os Draconis | 30 | 20090064 |
| Shou Wu Teng | Caulis Polygoni Multiflori | 15 | 20100033 |
| Suan Zao Ren | Semen Ziziphi Spinosae | 15 | 20120104 |
| Bie Jia | Carapax Trionycis | 9 | 20100457 |
| Fo Shou | Fructus Citri Sarcodactylis | 9 | 21020006 |
| Dan Shen | Salvia miltiorrhiza Bunge | 15 | 20100114 |
| He Huan Hua | Albizzia julibrissin Durazz | 9 | 20050143 |

**Supplementary material 2：**

The compositions of Hekun decoction.
