# Supplementary material for: Hekun decoction versus Femoston for women with amnestic mild cognitive impairment in early menopause: a randomized, three-arm, double-blind clinical trial
Source: Front Neurol. 2025 Sep 12;16:1610562. doi: 10.3389/fneur.2025.1610562 (PMC12463637; doi:10.3389/fneur.2025.1610562)
Supplement: Supplementary file 3 [file Table_3.docx]

**Supplementary material 3:**

The detailed reasons for withdrawal and exclusion of participants.

| **Case number** | **Exclusion time** | **Reason** |
| --- | --- | --- |
| 1 | Exclusion (Cycle 6) | Incorrect treatment allocation |
| 5 | Exclusion (Cycle 5) | Non-compliance with medication regimen |
| 6 | Exclusion (Cycle 6) | Failure to meet inclusion criteria |
| 7 | Exclusion (Cycle 6) | Non-compliance with medication regimen |
| 8 | Exclusion (Cycle 6) | Non-compliance with medication regimen |
| 9 | Exclusion (Cycle 6) | Violation of protocol regarding concomitant medication |
| 10 | Exclusion (Cycle 6) | Non-compliance with medication regimen |
| 11 | Exclusion (Cycle 4) | Violation of protocol regarding concomitant medication |
| 51 | Exclusion (Cycle 3) | Non-compliance with medication regimen |
| 78 | Exclusion (Cycle 6) | Non-compliance with medication regimen |
| 258 | Exclusion (Cycle 6) | Non-compliance with medication regimen |
| 307 | Exclusion (Cycle 6) | Non-compliance with medication regimen |
| 2 | Withdrawal (Cycle 3 ) | **Trial Discontinuation (Worsening of hot flashes, night sweats, palpitations, and insomnia)** |
| 3 | Withdrawal (Cycle 3 ) | **Trial Discontinuation (Exacerbation of hot flashes and night sweats)** |
| 4 | Withdrawal (Cycle 5) | **Trial Discontinuation (Aggravation of gastric distension and pain)** |
| 13 | Withdrawal (Cycle 4) | **Lost to Follow-up** |
| 15 | Withdrawal (Cycle 3 ) | **Lost to Follow-up after post-COVID-19 treatment** |
| 18 | Withdrawal (Cycle 4) | **Lost to Follow-up** |
| 19 | Withdrawal (Cycle 3 ) | **Lost to Follow-up** |
| 20 | Withdrawal (Cycle 3 ) | **Withdrawal** |
| 22 | Withdrawal (Cycle 5) | **Lost to Follow-up** |
| 67 | Withdrawal (Cycle 6) | **Withdrawal** |
| 199 | Withdrawal (Cycle 6) | **Lost to Follow-up** |
| 206 | Withdrawal (Cycle 6) | **Lost to Follow-up** |
| 210 | Withdrawal (Cycle 4) | **Lost to Follow-up** |
| 212 | Withdrawal (Cycle 1) | **Lost to Follow-up** |
| 214 | Withdrawal (Cycle 4) | **Lost to Follow-up** |
| 215 | Withdrawal (Cycle 4) | **Lost to Follow-up** |
| 216 | Withdrawal (Cycle 4) | **Lost to Follow-up** |
| 217 | Withdrawal (Cycle 4) | **Lost to Follow-up** |
| 248 | Withdrawal (Cycle 4) | **Lost to Follow-up** |
| 249 | Withdrawal (Cycle 6) | **Lost to Follow-up** |
| 275 | Withdrawal (Cycle 1) | **Lost to Follow-up** |
| 280 | Withdrawal (Cycle 3 ) | **Lost to Follow-up** |
| 281 | Withdrawal (Cycle 3 ) | **Lost to Follow-up** |
| 291 | Withdrawal (Cycle 3 ) | **Trial Discontinuation (Exacerbation of hot flashes and night sweats)** |
| 453 | Withdrawal (Cycle 6) | **Trial Discontinuation (Worsening of epigastric pain)** |
