# Supplementary material for: Hekun decoction versus Femoston for women with amnestic mild cognitive impairment in early menopause: a randomized, three-arm, double-blind clinical trial
Source: Front Neurol. 2025 Sep 12;16:1610562. doi: 10.3389/fneur.2025.1610562 (PMC12463637; doi:10.3389/fneur.2025.1610562)
Supplement: Supplementary file 4 [file Table_4.DOCX]

# Supplementary material 4：

# The details of adverse events

| Adverse Events | No. (%) |  | |
| --- | --- | --- | --- |
|  | **Hekun decoction**  （*n* = 98） | **Femoston**  (*n* = 98) | **Placebo**  （*n* = 96） |
| Digestive system |  |  |  |
| Abdominal distension and pain | 0 (0.00) | 5 (5.10) | 1 (1.04) |
| Diarrhea | 1 (1.02) | 0 (0.00) | 1 (1.04) |
| Reproductive System |  |  |  |
| Breast pain | 0 (0.00) | 2 (2.04) | 0 (0.00) |
| Abnormal uterine bleeding | 0 (0.00) | 3 (3.06) | 0 (0.00) |
| Respiratory System |  |  |  |
| Common cold | 1 (1.02) | 6 (6.12) | 0 (0.00) |
| Nervous System |  |  |  |
| Increased hot flashes | 1 (1.02) | 0 (0.00) | 2 (2.08) |
| Worsened dizziness | 1 (1.02) | 1 (1.02) | 1 (1.04) |
| Worsened insomnia | 0 (0.00) | 0 (0.00) | 1 (1.04) |
| Worsened fatigue | 0 (0.00) | 1 (1.02) | 1 (1.04) |
| Worsened lower back pain | 0 (0.00) | 1 (1.02) | 0 (0.00) |
| Musculoskeletal System |  |  |  |
| Worsened muscle pain | 0 (0.00) | 0 (0.00) | 2 (2.08) |
| Total | 4 (4.08) | 19 (19.39) | 9 (9.38) |
